# Clinical diagnosis and treatment of scabies, a neglected tropical disease

**DOI:** 10.4102/safp.v63i1.5224

**Published:** 2021-07-09

**Authors:** Hendrick M. Motswaledi

**Affiliations:** 1 Department of Dermatology, Faculty of Health Sciences, Sefako Makgatho Health Sciences University, Tshwane, South Africa

**Keywords:** scabies, *Sarcoptes scabiei*, permethrin, ivermectin, non-fatal disease, pyoderma

## Abstract

Scabies is a parasitic infestation of the skin caused by the mite *Sarcoptes scabiei* var. *hominis*. It is common in tropical areas, including the sub-tropical areas of Southern Africa. Predisposing factors are overcrowding, poor personal hygiene, low socio-economic standards and impaired host immunity. Although it can occur at any age, scabies is commonly seen in children and young adults. It is not a fatal disease; however, it can cause severe morbidity and poor quality of life. Scabies can complicate with pyoderma which may result in post-streptococcal glomerulonephritis. There are two clinical variants, classic scabies and the much rarer crusted scabies (Norwegian scabies).

## Classic scabies

The term ‘scabies’ is derived from the Latin word ‘scabere’ meaning to scratch.^1^ Scabies is a parasitic infestation caused by the mite *Sarcoptes scabiei* var. *hominis.*^2^ This mite is a host specific, obligate parasite in humans.^3,4^

In 2013, scabies was recognised by the World Health Organization (WHO) as one of the neglected tropical diseases of public health importance.^5,6^ It is estimated to affect more than 130 million people globally at any time.^5,6^

## Epidemiology

It is a worldwide problem affecting all races and socio-economic groups.^3^ It occurs commonly in children, but can occur at any age.^2^ Several members of a family can be simultaneously affected as the mites are transmitted by close personal contact.^1^

It is common in poor communities because of overcrowded living conditions, for example, in old age homes and prisons as well as in homeless and displaced children.^7^ In developed countries, it can occur as small epidemics in situations like war and during natural disasters.^1,2^

Once the human skin is infested, female mites burrow into the stratum corneum of the epidermis. They live for 4 to 6 weeks and produce two to four eggs per day, deposited into the burrowed tunnel. Larvae hatch 4 days later and develop into adult mites after 10 to 14 days.^8^ Mites can live for up to 3 days without a human host and there is evidence that fomites like clothing and linen can spread classic scabies.^9^

## Clinical presentation

Pathognomonic lesions are burrows that are caused by the mite as it penetrates the skin. The mite produces a lytic secretion which dissolves the host tissue and allows the mite to propel itself forward in the stratum corneum.^1,2,3^ Clinical manifestations of scabies typically develop 3–4 weeks after infestation. Clinical features are mainly because of an immune response to the presence of mites in the skin.^1,4^ It should be noted that the development of an immune response does not eliminate the disease and does not confer immunity against reinfestation.^1^

Scabies is characterised by intense pruritus, often worse nocturnally.^1,3^ Pruritus is the result of a hypersensitivity reaction to components of the saliva, ova and faecal material of the mites.^10^

Cutaneous lesions are inflammatory papules, vesicles, indurated nodules and pustules. Scabies can mimic many other skin diseases such as atopic eczema, seborrhoeic dermatitis, disseminated varicella and many others.

**FIGURE 1:**
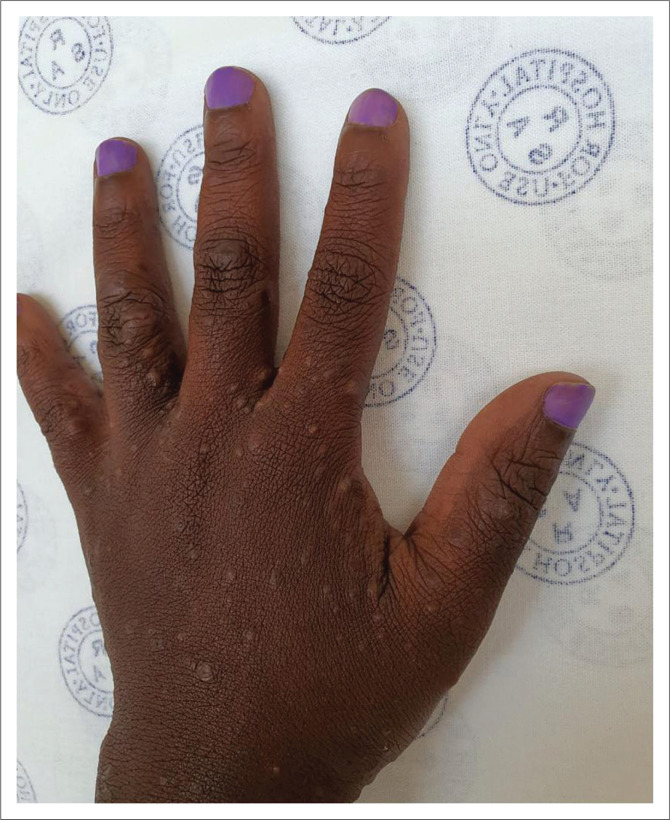
Scabies papules on the dorsum of the hand, note the involvement of the web spaces.

**FIGURE 2:**
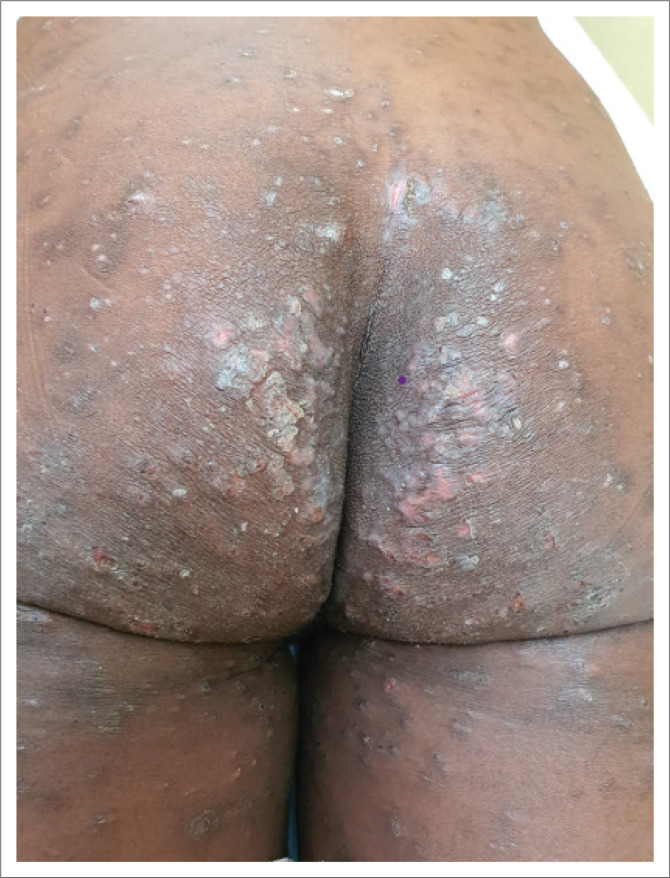
Eczematisation of scabies on thighs, buttocks and intergluteal cleft. Note the excoriations and crusting.

Common areas for scabies lesions are the flexor aspect of wrists, dorsal aspects of hands (Figure 1), finger web spaces, palms, sides of fingers, feet, axillae, umbilicus and intergluteal cleft (Figure 2).^1,2,3^ Male genitalia and areolae in females are commonly affected.

In neonates, scabies may be difficult to diagnose as the clinical picture is different to that of older children and adults. In this age group, the eruption turns to be generalised, including head, neck, face, palms and soles with early tendency to pustule formation.^11^

## Complications

Complications of scabies include eczematisation, secondary bacterial infections and post-streptococcal glomerulonephritis. Long-standing lesions can have secondary changes like eczematisation that is widespread and resembles chronic eczema. Scratching and excoriations cause breaks in the epidermis, serving as entry points for pathogenic bacteria. Secondary infection of scabies lesions may result in folliculitis, impetigo, ecthyma and abscesses. If secondary infection with β-haemolytic *Streptococci* or nephritogenic strains of *Streptococcus pyogenes* occurs, this may result in post-streptococcal glomerulonephritis.^5,10,12^ This is presumably by way of molecular mimicry. Studies have shown that epidemics of scabies have resulted in increased incidence of post-streptococcal glomerulonephritis in some populations.^13^

## Diagnosis

**FIGURE 3:**
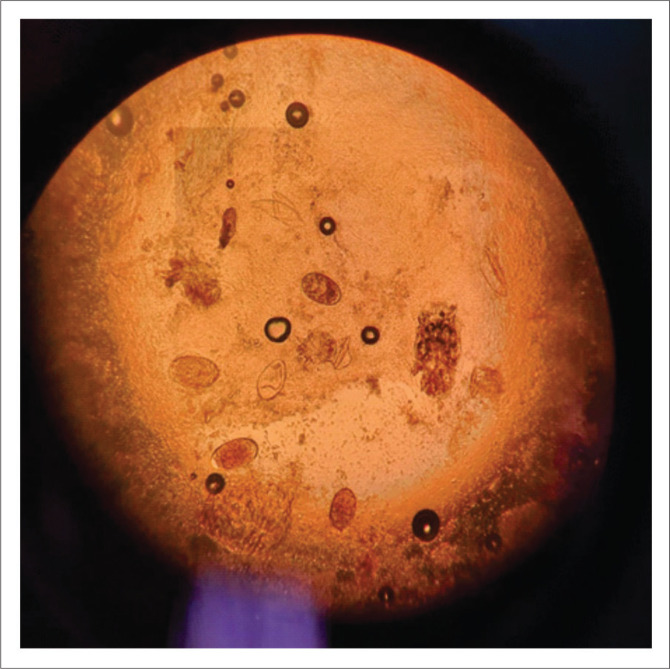
Ova of *Sarcoptes scabiei* on potassium hydroxide preparation.

Scabies is confirmed by detecting the mites, ova and faecal matter with microscopy. A skin scraping of a burrow is done with a scalpel blade, and the material is put on a glass slide; 10% potassium hydroxide (KOH) or mineral oil is added, and the slide is examined microscopically (Figure 3).^1,2,3,4^

This, however, is not always possible, especially in classic scabies, where the mite burden is lower. In this situation, it is acceptable to presume the diagnosis based on history and clinical findings.

Dermoscopy can also be performed using a hand-held dermatoscope with x10 magnification, but this has a low specificity. Skin biopsy is only done for difficult cases and to rule out other skin diseases like chronic eczema and psoriasis.

## Treatment

Treatment of scabies is aimed at elimination of the mites, treatment of symptoms and signs, as well as treatment of secondary bacterial infection if present.

### Prevention of spread

Close house-hold and family contacts must all be treated to help prevent the spread of the disease and also to prevent reinfection.

Bed linen, towels and clothing must be soaked in boiling water, and washed and dried in the sun to eliminate the mites.

### Topical treatment

Scabicidal soaps like Tetmosol soap^®^ (which contains monosulfirum 5%) should be used for bathing. Permethrin 5% lotion or cream is the first-line treatment of scabies in many countries. Permethrin acts on the nerve cell membranes of the mites to disrupt the sodium channel current by which the polarisation of the membrane is regulated. This results in delayed polarisation and subsequent paralysis and death of the mites.

Permethrin 5% is applied once at night and washed off in the morning. Re-application is always recommended, because mite eggs hatch 2–6 days later and can result in disease recurrence. It is effective, has low toxicity and is less irritant to the skin than benzyl benzoate and therefore suitable for use in children.^10,14,15^ Its safety in pregnancy and lactation has not been established. In addition to concerns over toxicity of these topical compounds, parasite resistance seems to be increasing.^9^

Permethrin is not always available in primary healthcare setting in South Africa. Benzyl benzoate is recommended on day 1, day 2 (2 consecutive days) and day 7. In paediatrics, a 10% formulation is available as opposed to the 25% adult formulation that can irritate the immature skin more.

Benzyl benzoate is a strong skin irritant which may cause a burning sensation, resulting in reduced compliance. Children may not tolerate it. A 10% sulphur mixed with petrolatum base or emulsifying ointment can be used and is well tolerated by children. Sulphur is messy and malodorous and can stain clothes and linen, but its efficacy in treating scabies is high. When applying topical agents, specific attention should be paid to the finger nails, toe nails, post-auricular areas, groins, interdigital spaces, natal cleft and axillary regions. Scabies patients can experience itch up to a month after successful treatment. Second-generation antihistamines may be necessary for a month to alleviate the itch. Adjuvant treatment like chlorhexidine antiseptics may be necessary to prevent secondary bacterial infection.

## Crusted scabies (Norwegian scabies)

Crusted scabies, formerly called Norwegian scabies, was first described in 1848 in leprosy patients in Norway.^1,4^ It is a type of scabies in which a large number of mites are present in the skin, and thus, the host’s response gets overwhelmed, allowing the mites to multiply. There are millions of mites in a patient with crusted scabies.^16^

Crusted scabies often occurs in patients who are immunocompromised, for example, patients infected with HIV, human T-cell lymphotropic virus-1 or leukaemia, neurologically impaired patients who cannot scratch, for example, paraplegics and quadriplegics, as well as in leprosy patients with neuropathies.^16^ It may also occur in patients with mental disorders like senile dementia, Down’s syndrome^17^ and patients with severe nutritional deficiencies.^1,3,4^ It is rarely seen in patients with healthy immune systems.^10,12,16^

**FIGURE 4:**
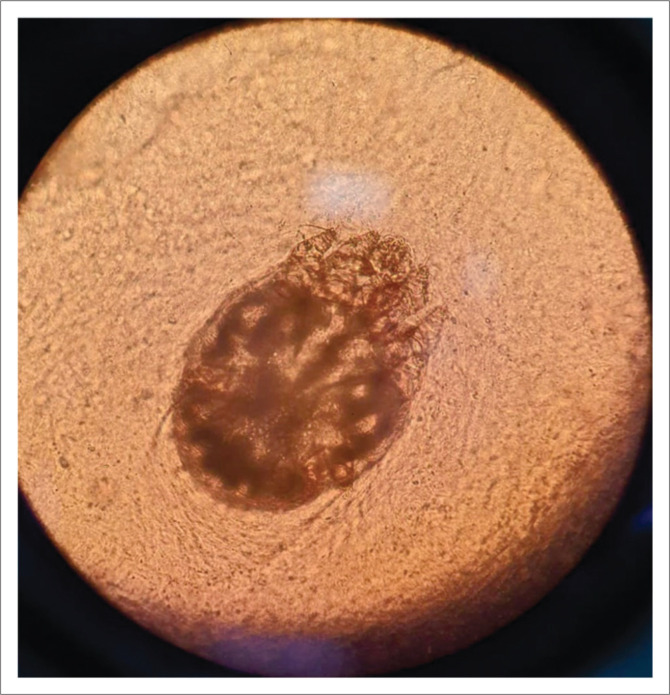
*Sarcoptes scabiei* mite on potassium hydroxide preparation.

**FIGURE 5:**
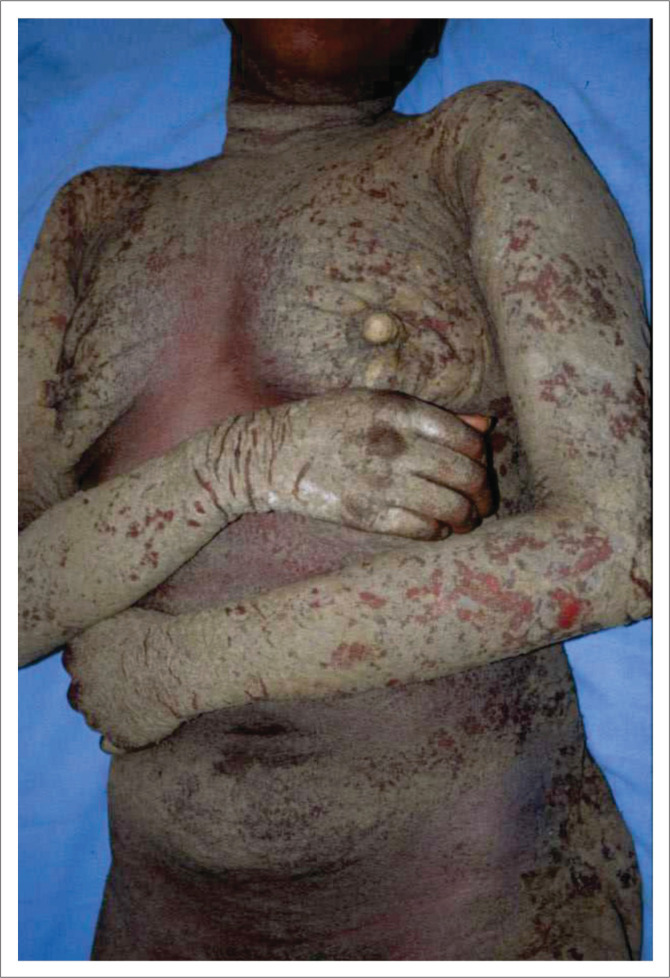
Crusted scabies in an immunocompromised patient.

**FIGURE 6:**
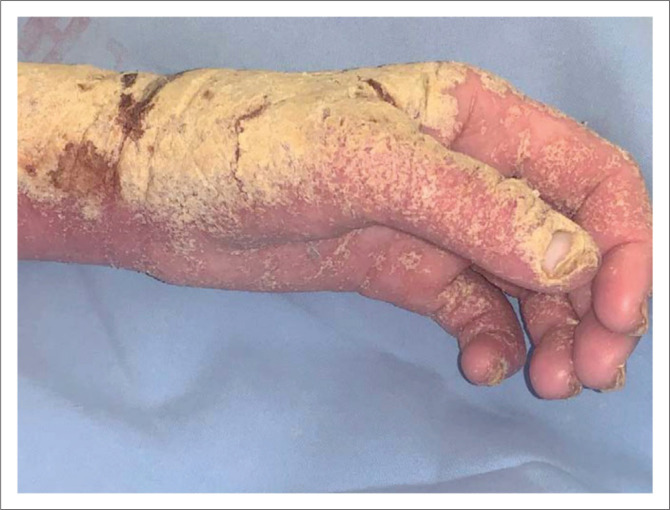
Crusted scabies in an albino patient, note the subungual hyperkeratosis.

In crusted scabies, thick hyperkeratotic crusts occur on the skin with erythroderma (Figures 4 and 5). This clinical picture can resemble psoriasis.^17^ The crusts carry millions of these mites and may shed them off to the patient’s environment, thereby infecting others. Unlike in classic scabies, in crusted scabies, a skin biopsy is almost always diagnostic as mites and ova can be easily visualised in the thickened stratum corneum (Figure 6). Peripheral eosinophilia and immunoglobulin E levels are usually raised in patients with crusted scabies.^8,10,18^

**FIGURE 7:**
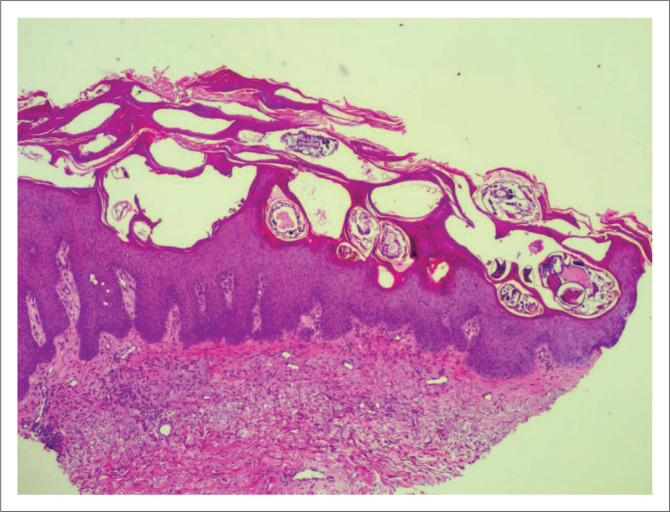
Honeycombed stratum corneum with scabies mites and ova (magnification x40).

Treatment for crusted scabies is the same as for ordinary scabies, but multiple applications of scabicides are required (Figure 7). Ideally, patient with crusted scabies must be admitted in hospital isolation ward to treat underlying conditions as well. Ivermectin if available can be used in recalcitrant cases.

Oral ivermectin, a macrocyclic lactone structurally similar to the macrolide antibiotics but devoid of antibacterial activity, has a good safety profile^19,20^ and has revolutionised treatment of ectoparasites.^21,22^ The efficacy of oral ivermectin is equivalent to or better than that of topically applied lindane^23^ and benzyl benzoate.^24^ In one study, two doses of ivermectin were found to be as effective as a single application of permethrin.^25^ Oral ivermectin can also eliminate intestinal parasites and therefore can benefit patients who are polyparasitised with enteroparasites and ectoparasites.^19,26^

**FIGURE 8:**
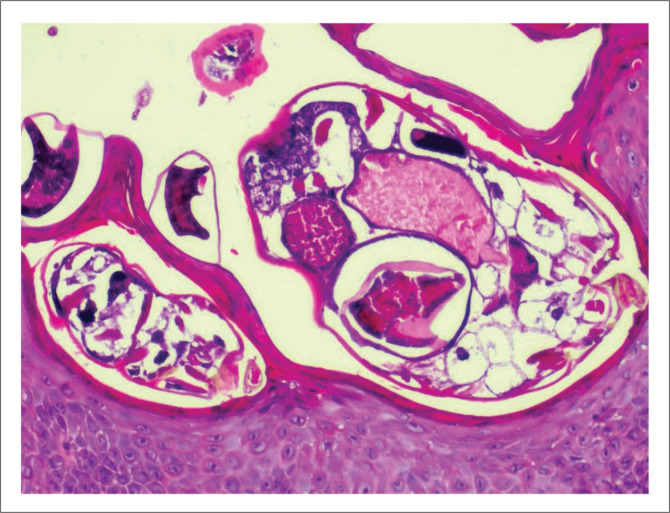
Higher magnification showing scabies mites (magnification x100).

**FIGURE 9:**
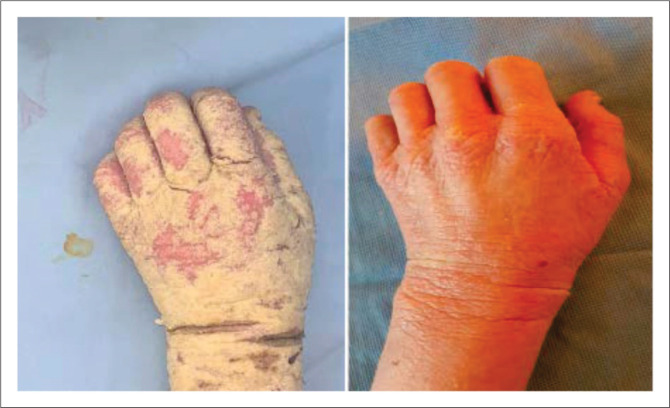
Crusted scabies before and after treatment. This patient was treated with permethrin 5% lotion, two applications a week apart.

Oral ivermectin in single doses of 200 μg/kg, with a second dose given after 10 days, has been shown to be very effective in a number of studies.^26,27^ Ivermectin is very useful in controlling the outbreaks of scabies in closed communities such as prisons where it is easy to administer a single dose under supervision and avoid problems of compliance and inadequate application associated with topical therapy.^19^

Ivermectin is also useful in treating patients with scabies and HIV and other immunocompromised patients, who may be difficult to cure as they require several applications of different topical agents or combinations of therapy over a period of weeks.^28^

In many African countries where parasitic infestations like scabies, onchocerciasis and filariasis are a major problem, patients are treated with intramuscular or oral ivermectin.^29^ In South Africa, currently injectable ivermectin is only registered for veterinary use.^30^

## Conclusion

Scabies is a fairly common skin disease. Although it is not a fatal disease, it may result in severe morbidity and poor quality of life. Clinicians must acquaint themselves with its diagnosis and treatment.
